# Textbook Oncological Outcomes for Robotic Colorectal Cancer Resections: An Observational Study of Five Robotic Colorectal Units

**DOI:** 10.3390/cancers15153760

**Published:** 2023-07-25

**Authors:** José Moreira Azevedo, Sofoklis Panteleimonitis, Danilo Mišković, Ignacio Herrando, Mahmood Al-Dhaheri, Mukhtar Ahmad, Tahseen Qureshi, Laura Melina Fernandez, Mick Harper, Amjad Parvaiz

**Affiliations:** 1 Champalimaud Foundation, Av. Brasilia, 1400-038 Lisbon, Portugallaura.fernandez@fundacaochampalimaud.pt (L.M.F.); apcheema@yahoo.com (A.P.); 2 Faculty of Medicine, University of Lisbon, Av. Prof. Egas Moniz MB, 1649-028 Lisbon, Portugal; 3 School of Health and Care Professions, University of Portsmouth, St. Andrews Court, St. Michael’s Road, Portsmouth PO1 2PR, UK; mick.harper@port.ac.uk; 4 St. Mark’s Hospital, London NW10 7NS, UK; danilo.miskovic@nhs.net; 5 Hamad General Hospital, Doha 3050, Qatar; maldhaheri14@gmail.com; 6 Poole Hospital NHS Trust, Longfleet Road, Poole BH15 2JB, UK; mukhtardatti@yahoo.co.uk (M.A.); tasqureshi007@gmail.com (T.Q.)

**Keywords:** robotic surgery, colorectal cancer, surgical outcomes, colon cancer, rectal cancer, quality of care

## Abstract

**Simple Summary:**

The quality of care of patients receiving colorectal resections has conventionally relied on individual metrics. When discussing with patients what these outcomes mean, they often find them confusing or overwhelming. Textbook outcomes are a composite measure that summarises all the ‘desirable’ clinical and oncological outcomes. This study aims to evaluate the incidence of textbook outcomes in patients receiving robotic colorectal cancer surgery. We present a retrospective, multicentric study with data from a prospectively collected database. A textbook outcome was achieved when all components were realized: no conversion to open, no complication with a Clavien–Dindo ≥ 3, length of hospital stay ≤ 14, no 30-day readmission, no 30-day mortality, and R0 resection. Nearly 80% of patients achieved a textbook outcome, and abdominoperineal resection was a risk factor for failure. The rate of a textbook outcome may be used in future audits and to inform patients clearly on the success of treatment.

**Abstract:**

Background: The quality of care of patients receiving colorectal resections has conventionally relied on individual metrics. When discussing with patients what these outcomes mean, they often find them confusing or overwhelming. Textbook oncological outcome (TOO) is a composite measure that summarises all the ‘desirable’ or ‘ideal’ postoperative clinical and oncological outcomes from both a patient’s and doctor’s point of view. This study aims to evaluate the incidence of TOO in patients receiving robotic colorectal cancer surgery in five robotic colorectal units and understand the risk factors associated with failure to achieve a TOO in these patients. Methods: We present a retrospective, multicentric study with data from a prospectively collected database. All consecutive patients receiving robotic colorectal cancer resections from five centres between 2013 and 2022 were included. Patient characteristics and short-term clinical and oncological data were collected. A TOO was achieved when all components were realized—no conversion to open, no complication with a Clavien–Dindo (CD) ≥ 3, length of hospital stay ≤ 14, no 30-day readmission, no 30-day mortality, and R0 resection. The main outcome measure was a composite measure of “ideal” practice called textbook oncological outcomes. Results: A total of 501 patients submitted to robotic colorectal cancer resection were included. Of the 501 patients included, 388 (77.4%) achieved a TOO. Four patients were converted to open (0.8%); 55 (11%) had LOS > 14 days; 46 (9.2%) had a CD ≥ 3 complication; 30-day readmission rate was 6% (30); 30-day mortality was 0.2% (1); and 480 (95.8%) had an R0 resection. Abdominoperineal resection was a risk factor for not achieving a TOO. Conclusions: Robotic colorectal cancer surgery in robotic centres achieves a high TOO rate. Abdominoperineal resection is a risk factor for failure to achieve a TOO. This measure may be used in future audits and to inform patients clearly on success of treatment.

## 1. Introduction

Quality of care assessment and audit of patients receiving colorectal cancer resections has conventionally relied on individual metrics such as length of stay (LOS), clear resection margin, 30-day readmission, and 30-day mortality [1,2,3]. Surgeons are increasingly aware of their duty to inform patients of the quality of care they provide. However, patients have indicated that they prefer summarised metrics over detailed individual outcomes when being informed about the quality of care they receive [4]. Textbook Oncological Outcome (TOO) is a composite measure that summarises all the ‘desirable’ or ‘ideal’ postoperative clinical and oncological outcomes from both a patient’s and doctor’s point of view [5,6]. It was introduced as a merged measurement reflecting average ‘best’ surgical quality [5,7] and has several advantages over single outcome variables, such as the ability to summarise performance and prevent indicator-driven practice [5,8].

Over the last decade, robotic surgery has played an ever-increasing role in colorectal cancer surgery, evident from the increasing number of studies published on the subject [9,10,11]. The National Bowel Cancer (NBOCA) audit from England and Wales has shown a sharp increase on the number of robotic colorectal resection performed from 239 to 565 in a space of four years [12] resulting in multiple studies examining the short-term clinical and oncological outcomes of robotic colorectal cancer surgery with its safety and feasibility reported as being well stablished [3,9,10,11,13]. Nevertheless, when discussing with patients in the outpatient setting what these outcomes mean, they often find them confusing or overwhelming indicating that a more useful summarised, composite outcome that is easier for patients to understand and can be used as a benchmark for future audits is required.

There are few studies examining the TOO of patients receiving colorectal cancer surgery [5,6,7,8,14,15]. However, as far as we are aware, there are no studies examining the TOO of robotic colorectal cancer resections. Therefore, it would be interesting to investigate how robotic colorectal centres perform in terms of TOO. To answer this question, this study aims to evaluate the incidence of TOO in patients receiving robotic colorectal cancer surgery in five robotic colorectal units and investigate the risk factors associated with failure to achieve a TOO in such patients.

## 2. Materials & Methods

A retrospective analysis of prospectively maintained databases was conducted for this study. Consecutive cases from five robotic colorectal cancer units, three from the UK (St Mark’s, Portsmouth, and Poole), one from Portugal (Champalimaud Foundation) and one from Qatar (Hamad General Hospital, Doha) who received robotic colorectal cancer resections between 2013 and 2022 were identified and included in this study. The inclusion criteria were all patients with colon or rectal cancer receiving elective robotic resection. Benign cases or cases missing data on any one of the variables used to define a TOO were excluded. This resulted in a sample of 501 patients.

All cancer patients involved in this study were discussed in the multidisciplinary team meeting (MDT) prior to initiating any type of treatment. Neo-adjuvant treatment was given according to local guidelines following MDT discussion, and patients were prepared for surgery according to each regional institution’s policy. In general, preoperative chemoradiotherapy was administered to patients with high risk for local recurrence (threatened circumferential resection margin ≤ 2 mm or T4 in staging MRI) or at the Champalimaud Foundation also in patients with extramural venous invasion (EMVI). Node status was not a criterion for neoadjuvant treatment in any of the participating units. Patients receiving neoadjuvant chemoradiotherapy were operated at 12 weeks after completion of their treatment or 6 months after treatment if they received consolidation chemotherapy. A modified enhanced recovery programme was used as standard at all colorectal units in this study [16].

All patients operated in Portsmouth received surgery with the da Vinci Si robotic system™. Patients operated in the remaining units received surgery with the da Vinci Xi robotic system™. Robotic surgeries were performed by five experienced robotic colorectal surgeons. A fully robotic single docking technique was applied for all surgeries, following the principle of dissection through the embryological planes as described in previously published research [17,18,19].

The requirements for anonymization of personal dataset by the Data Protection Act 1998 were satisfied. According to the Health Research Authority (HRA), this study did not require their approval due to its status as a clinical audit. However, approval according to local regulations was followed with the need for ethical committee submission at Champalimaud Foundation.

### 2.1. Data Collection and Outcome Assessment

All data were collected from a prospectively maintained databases from each institution. Baseline characteristics analysed included age, body mass index (BMI), gender, American Society of Anaesthesiologist (ASA) grade, neoadjuvant radiotherapy, operation performed, distance to anal verge (for rectal cancer only), and pathological T and N stage. Short-term clinical and oncological data included conversion to open (defined as any incision needed to either mobilise the colon or rectum or ligate the vessels), LOS, post-operative complications with Clavien–Dindo (CD) ≥ 3 score [20], 30-day readmission, 30-day mortality, lymph node yield, and resection margin clearance.

A subgroup analysis was performed for patients receiving robotic rectal or colon resections to investigate the incidence of TOO for rectal and colon resections as a separate group.

### 2.2. Textbook Oncological Outcome (TOO)

The parameters defining a TOO were agreed upon with all the co-authors, taking into consideration previous published studies reporting TOO in colon and rectal cancer surgery [5,6,7,8,14,15]. A TOO was achieved when all components were realised and is expressed as a percentage. The parameters were no conversion to open, no complication Clavien–Dindo (CD) ≥ 3, LOS ≤ 14, no 30-day readmission, no 30-day mortality, and R0 resection. 

### 2.3. Statistical Analysis

Data were analysed using an IBM SPSS version 26 (SPSS Inc., Chicago, IL, USA). The proportion of patients achieving a TOO is presented as a percentage, and the proportion of cases meeting each individual criterion for a TOO is also presented. In addition, we present the cumulative percentages of patients in whom each consecutive outcome was met, under the condition that all previous conditions are met. This is accordance with previously published reports [6,7,8]. For logistic regression analysis, missing values were replaced with multiple imputations of the SPSS Impute Missing Data Values function. Missing values are presented in a Supplementary Table S1. Five imputations were created, and the maximum number of case draws was set to 200.

The baseline characteristics of patients achieving a TOO vs. those that did not are analysed to identify any risk factor associated with failure to achieve a TOO. Non-parametric data were expressed as median with interquartile range, and parametric data were expressed as mean with standard deviation. Characteristics were compared using the χ^2^ test or Fishers exact test for categorical variables, Mann–Whitney U test for non-parametric continuous variables, and the *t* test for parametric continuous variables. We have purposely avoided using the term statistical significance in accordance with the latest movement against applying this term [21].

Finally, a univariate logistic regression analysis was performed to assess whether any baseline characteristics affected TOO. Variables with *p* < 0.300 on univariate analysis were entered on multivariate analysis. The constant was included in the analysis model, and data are presented as odds ratio, 95% confidence interval, and *p* value.

## 3. Results

A total of 501 patients receiving elective robotic colorectal cancer resections between 2013 and 2022 were identified. 

### 3.1. Textbook Oncological Outcome (TOO)

**Figure 1:**
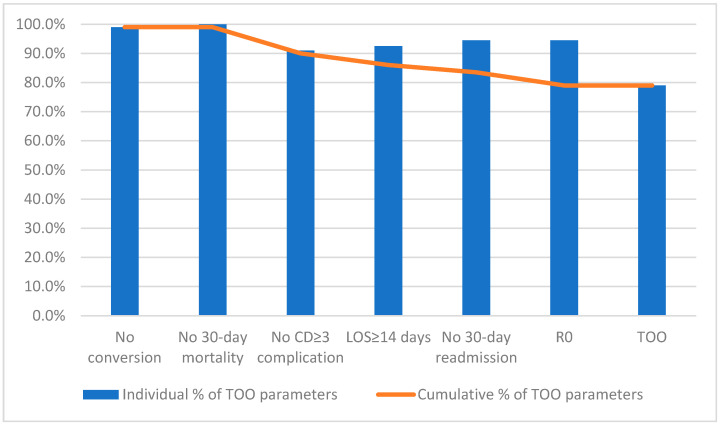
The rates of the individual TOO parameters (bars) and cumulative percentage (line) of TOO after each parameter.

Of the 501 patients that underwent robotic colorectal cancer surgery, 388 (77.4%) achieved a TOO. Four patients were converted to open (0.8%); 55 patients (11%) had LOS > 14 days; 46 patients (9.2%) had a CD ≥ 3 complication; 30-day readmission rate was 6% (30 patients); 30-day mortality was 0.2% (1 patient); and 480 patients (95.8%) had an R0 resection. Table 1 summarizes these findings and shows the cumulative percentages of each parameter, which are also illustrated on Figure 1.

### 3.2. Cohort Characteristics

Table 2 summarizes the cohort characteristics of the patients who achieved and did not achieve a TOO. There were differences in the percentages of procedures performed between the two groups (*p* = 0.024), and more notably there were twice as many robotic APER’s in the group that did not achieve TOO (18.6% vs. 9.5%). For rectal resections, the distance between the anal verge was lower in the group that did not achieve a TOO, 7.00 cm (5.00–9.00) vs. 8.50 cm (5.45–13.70), *p* = 0.015. The remaining cohort characteristics were similar between the two groups. There is no statistical difference (chi-square) in terms of hospitals for TOO. In multivariate regression analysis, it does not affect the other outcomes.

### 3.3. Rectum vs. Colon Robotic Resections Subgroup Analysis

Table 3 summarises the TOO and individual TOO parameters for patients receiving colon vs. rectum robotic resections. Rectal cancer resections were performed in 397 patients while 104 patients received colon cancer resections. TOO was achieved in 80.8% of colon resections and 76.6% of rectal resections (*p* = 0.362). There were three conversions in the colon group (2.9%) and one in the rectum group (0.3%), *p* = 0.030. A description of complications with a Clavien Dindo ≥ 3 are presented in Table 4.

### 3.4. Logistic Regression Analysis

Univariate logistic regression analysis showed APER was a risk factor for not achieving a TOO (OR 0.462, 95% CI 0.258–0.827, *p* = 0.009). This was still the case in multivariate analysis (OR 0.400, 95% CI 0.209–0.764, *p* = 0.006) when all parameters with a *p* < 0.300 were entered in the logistic regression analysis model (sex, BMI, and APER). None of the other baseline characteristics investigated were found to influence TOO. The above findings are summarised on Table 5.

## 4. Discussion

Currently there is a trend to examine textbook outcomes of patients receiving colorectal cancer surgery. To the authors’ knowledge, this is the only study to investigate how robotic colorectal cancer units perform in terms of TOO. Here, we present a multicentric retrospective study with the use of prospectively collected data that aims to assess the quality of surgical care with the gathering of a set of parameters called TOO. This evaluates the care with the success achieved for the total of the parameters included in the composite not with an individual parameter. TOO was defined as no conversion to open, no Clavien–Dindo (CD) complication ≥ 3, LOS ≤ 14, no 30-day readmission, no 30-day mortality, and R0 resection, in line with previous published reports [5,6,7,8,14,15].

Our data show that the specialized centres in robotic colorectal resections achieved a 77.4% rate of TOO. This rate reflects specialized care in these centres but also reflects the more complex patients referred to these units. The rate of advanced tumours T3/4 was almost half of the patients 44.7% (224). Likewise, although the study presents the TOO for colorectal surgery, the truth is that the use of robotic surgery ends up happening more routinely for patients with rectal cancer. Therefore, only 20.8% (104) of the patients presented were submitted to colon surgery. The remaining majority receiving APERs and anterior resections. Knowing that surgery of the rectum is more technically demanding and carries higher risks, both in the short term and in terms of oncological parameters, this TOO rate presented here reflects a high value for more demanding and complex patients.

Each of the parameters had its own impact on the likelihood of a patient obtaining a TOO. The main factors preventing success were CD ≥ 3 post-op complications, which occurred in 46 patients (9.2%), and a LOS > 14 days, which occurred in 55 patients (11%). In fact, the use of a length of stay longer than 14 days was chosen by the authors according to the existing literature on textbook outcomes. A limitation of this choice would be that patients undergoing minimally invasive surgery are likely to be discharged earlier, and therefore we may be missing patients who had minor complications that resolved in less than 14 days. The parameters that had less impact on the score were conversion to open surgery (0.8%) and 30-day mortality (0.2%). These data reflect the technical experience of the surgical teams both through the need to convert to robotic surgery in a very low number of cases and the low mortality, demonstrating that there is experience in dealing with postoperative complications, even in patients with malignant disease. A limitation could have been the use of mortality at 30 days instead of 90 days. However, first the studies in which TOO have been used have used mortality at 30 days, and we intend to standardise the practice. Secondly, there was no difference in the 90 days of mortality in our cohort. Surprisingly, the 30-day readmission rate of 6% is within the lower limits presented in the literature in other studies involving teams experienced in minimally invasive colorectal resections [22]. Another parameter that could lead to another endpoint in the future, concerning the experience of surgical teams, is the ability of surgeons to manage complications: rate of failure to rescue.

In the analysis of risk factors for not achieving a TOO, this study has shown that patients undergoing APER had a lower independent chance of achieving a TOO when compared with the rest of the cohort. The authors conclude that this is understandable in patients undergoing APER, who have a higher risk of failure, have a higher LOS than others, and have a high risk of surgical wound complications. Even though wound complications may stay outside the group of patients with a Clavien–Dindo ≥ 3, what we detected was that some of these patients needed to be readmitted and therefore counted as a failure due to readmission. Similarly, in our data, patients submitted to APER failed to achieve a TOO mainly not to wound complications but due to R1 resections. This finding is comparable with the results presented by Naffouje et al. [7] in which patients undergoing APER or exenteration were linked to inferior short-term outcomes and to positive CRM. Also, in another study [6], it was identified that increasing age, race, increased T stage, and care at low-volume centres were risk factors in the multivariable analysis that independently diminished the probability of obtaining a TOO.

The increasing use of robotic systems, namely DaVinci platforms, by experienced teams in minimally invasive colorectal surgery has a benefit for patients. In a recent multicentre, randomised, controlled, superiority trial undertaken at 11 hospitals, the patients with middle and low rectal cancer treated with robotic surgery achieved better oncological quality of resection, with less surgical trauma and better postoperative recovery when compared to laparoscopic procedures [23]. With the knowledge of TOO and the use of the values presented here, we can communicate to patients, in a more informed, clear, and simple way, the expectations regarding the success of surgical treatment in specialized centres and the real benefits of performing the treatment of colorectal cancer with robotic systems.

Similarly, treatment of patients in specialised centres has been associated with a higher number of patients achieving a TOO. In the study presented here, both the use of robotic surgery and the treatment of these patients in tertiary centres ended up being reflected in a TOO above values presented in previous publications. The data presented may be further justification for the need to reorganise healthcare systems to create referrals of more complex cases to specialised centres.

This study has some limitations. Due to the retrospective nature, it was not possible to collect the same type of variables, nor in the same way, among all the participating centres. However, all participating centres had data collected for the parameters defined for the TOO, so although the lack of other variables may influence the risk factors for failing to obtain a TOO in the multivariate analysis, this lack of data would not modify the TOO value studied.

Another limitation is that TOO does not inform where the department or team will be failing nor where the care could improve. As such, it should be recommended that TOO not be used for the continuous assessment of surgical care. For a continuous assessment, variables should be evaluated independently to plan improvement strategies. Similarly, as previously reported [8] the TOO is a composite outcome that combines oncological outcomes with surgical or short-term outcomes. This combination may obscure worse oncological outcomes with excellent short-term outcomes or vice versa. Therefore, it is recommended that surgeons know the results of the individual parameters as well. Notwithstanding, the use of TOO as a composite of parameters allows better communication between centres and allows establishing a measure of success that may be used in future audits. 

## 5. Conclusions

Robotic colorectal cancer surgery in robotic colorectal units allows for the achievement of a high textbook outcome rate. This measure may be used to inform patients clearly on success of treatment and may allow a measure to be used in future audits. Extended resections, such as APER, maintain a higher risk of failure to achieve a TOO when compared with non-extended resections, even within specialized robotic colorectal units. It is recommended that further research be conducted to clarify which parameters and values are acceptable to provide effective and safe treatment for patients with colorectal cancer.

## Figures and Tables

**Table 1:** Individual and cumulative parameter percentages for TOO.

	Individual (n, %)	Cumulative (n, %)
Total population	501	
no conversion	497 (99.2%)	497 (99.2%)
No mortality	500 (99.8%)	496 (99.0%)
No CD ≥ 3 complication	455 (90.8%)	451 (90.0%)
LOS ≤ 14 days	446 (89.0%)	420 (83.8%)
No readmission	471 (94.0%)	403 (80.4%)
R0	480 (95.8%)	388 (77.4%)
TOO	388 (77.4%)	

**Table 2:** Cohort characteristics of patients achieving and not achieving a TOO. c—χ^2^ test, m—Mann–Whitney U test.

	No TOO	TOO Achieved	*p* Value
Age	66 (58–75.75)	68 (59–76)	0.951 m
BMI	26.8 (23.2–29.0)	27 (23.9–30)	0.203 m
Sex			
Male	76 (67.3%)	238 (61.3%)	0.252 c
Female	37 (32.7%)	150 (38.7%)	
ASA score			
1–2	61 (76.3%)	234 (77.5%)	0.815 c
3–4	19 (23.8%)	68 (22.5%)	
Neoadjuvant radiotherapy	19/84 (22.6%)	81/309 (26.2%)	0.502 c
Procedure name			
Anterior resection	70 (61.9%)	272 (70.1%)	0.024 c
APER	21 (18.6%)	37 (9.5%)	
Right hemicolectomy	13 (11.5%)	60 (15.5%)	
Left colectomy	2 (1.8%)	8 (2.1%)	
Transverse colectomy	0	2 (0.5%)	
Sigmoid colectomy	0	1 (0.3%)	
Panproctocolectomy	2 (1.8%)	3 (0.8%)	
Subtotal	5 (4.4%)	3 (0.8%)	
Hartman’s	0	2 (0.5%)	
Rectal cancer resection	93/113 (82.3%)	304/388 (78.4%)	0.362 c
Distance for anal verge in cm	7 (5–9)	8.5 (5.45–13.7)	0.015 m
Lymph nodes harvested	20 (3–54)	22 (6–64)	0.200
Lymph nodes positive	1.4 (0–27)	1.3 (0–39)	0.804
pT stage			
T0–2	34 (41%)	129 (42.4%)	0.810 c
T3–4	49 (59%)	175 (57.6%)	
pN stage			
N0	19 (27.9%)	65 (26.9%)	0.859 c
N1–2	49 (72.1%)	177 (73.1%)	

**Table 3:** TOO parameters for colon and rectal resections. c—χ^2^ test, m—Mann–Whitney U test, f—Fisher exact test.

	Colon (=104)	Rectum (n = 397)	*p* Value
Conversion	3 (2.9%)	1 (0.3%)	0.030 f
30-day mortality	1 (1%)	0	0.208 f
CD ≥ 3 complication	9 (7.8%)	37 (9.3%)	0.834 c
LOS > 14 days	8 (7.7%)	47 (11.8%)	0.229 c
LOS in days	5 (4–7)	5 (4–8)	0.215 m
30-day readmission	4 (3.8%)	26 (6.5%)	0.362 f
R0	102 (98.1%)	378 (95.2%)	0.274 f
TOO	84 (80.8%)	93 (76.6%)	0.362 c

**Table 4:** Description of complications CD ≥ 3.

Complications	n
Ileus with critical care admission	6
Small bowel occlusion	5
Parastomal hernia	2
Incisional hernia	2
Incarcerated inguinal hernia	1
Intra-abdominal collection	10
Anastomotic leak	12
Segmental mesenteric vein thromboses	1
Perforated diverticulum above anastomoses	1
Bleeding/Pelvic hematoma	3
Urosepsis	1
Pneumoniae	1
Arrhythmia + Pacemaker	1
Total	46

**Table 5:** Logistic regression for baseline characteristics effect on achieving TOO.

	Univariate				Multivariate			
	OR	95% CI Lower	95% CI Upper	*p* Value	OR	95% CI Lower	95% CI Upper	*p* Value
Age	1.001	0.983	1.020	0.880				
Sex (male)	1.295	0.831	2.016	0.253	1.293	0.828	2.019	0.259
BMI	1.029	0.973	1.088	0.315				
ASA grade (I–II vs. III–IV)	1.013	0.579	1.772	0.963				
Neoadjuvant RT	1.095	0.622	1,927	0.750				
Rectal cancer	0.778	0.453	1.336	0.363				
pT stage (T0–2 vs. T3–4)	1.071	0.609	1.885	0.805				
pN stage (N0 vs. N1–2)	1.097	0.566	2.126	0.774				
APER	0.462	0.258	0.827	0.009	0.462	0.258	0.829	0.010

## Data Availability

Data for this study are kept on a secure server in each of the enrolled institutions.

## Supplementary Materials

The following supporting information can be downloaded at: https://www.mdpi.com/article/10.3390/cancers15153760/s1, Table S1: Missing Values as percentage.
