# Supplementary material for: Textbook Oncological Outcomes for Robotic Colorectal Cancer Resections: An Observational Study of Five Robotic Colorectal Units
Source: Cancers (Basel). 2023 Jul 25;15(15):3760. doi: 10.3390/cancers15153760 (PMC10417291; doi:10.3390/cancers15153760)
Supplement: Supplementary file 1 [file cancers-15-03760-s001.zip › cancers-2388703-supplementary.pdf]

Supplementary Table S1. Missing values as percentage (%).

	**Missing**	
	**N**	**Percent**
Age	108	21.56%
BMI	133	26.55%
Sex	0	0.0%
Hospital	0	0.0%
ASA 1/2 vs 3/4	119	23.8%
RT (Y/N)	108	21.6%
Procedure	0	0.0%
Rectum	0	0.0%
Anal verge distance	177	44.58%
LN harvested	111	22.16%
LN +ve	239	47.7%
T1/2 vs T3/4	114	22.8%
N1/2	191	38.1%
Convert	0	0.0%
Death	0	0.0%
ClavienDindo ≥3	0	0.0%
LOS >14	0	0.0%
LOS	0	0.0%
Readmission	0	0.0%
R0/1	0	0.0%
